# A phloem-limited fijivirus induces the formation of neoplastic phloem tissues that house virus multiplication in the host plant

**DOI:** 10.1038/srep29848

**Published:** 2016-07-19

**Authors:** Jiangfeng Shen, Xian Chen, Jianping Chen, Liying Sun

**Affiliations:** 1 State Key Laboratory of Crop Stress Biology for Arid Areas and College of Plant Protection, Northwest A&F University, Yangling, Shaanxi, 712100, China; 2 Zhejiang Academy of Agricultural Sciences, Hangzhou 310021, P. R. China

## Abstract

A number of phloem-limited viruses induce the development of tumours (enations) in the veins of host plants, but the relevance of tumour induction to the life cycle of those viruses is unclear. In this study, we performed molecular and structural analyses of tumours induced by rice black-streaked dwarf virus (RBSDV, genus *Fijivirus*) infection in maize plants. The transcript level of the maize *cdc2* gene, which regulates the cell cycle, was highly elevated in tumour tissues. Two-dimensional electrophoresis identified 25 cellular proteins with altered accumulation in the tumour tissues. These proteins are involved in various metabolic pathways, including photosynthesis, redox, energy pathways and amino acid synthesis. Histological analysis indicated that the tumours predominantly originated from hyperplastic growth of phloem, but those neoplastic tissues have irregular structures and cell arrangements. Immunodetection assays and electron microscopy observations indicated that in the shoots, RBSDV is confined to phloem and tumour regions and that virus multiplication actively occurs in the tumour tissue, as indicated by the high accumulation of non-structural proteins and formation of viroplasms in the tumour cells. Thus, the induction of tumours by RBSDV infection provides a larger environment that is favourable for virus propagation in the host plant.

As obligate intracellular parasites, viruses exploit host cellular components to multiply and complete their life cycle^1^. Consequently, viral infection results in alteration of host growth and morphology through the modification of expression, translocation and functional activity of various host factors^2^. In plants, these alterations are manifested as various viral symptom phenotypes, such as necrosis, stunted growth, crinkled leaves and mosaic or chlorotic leaves^3^. Varieties of plant pathogenic agents can cause uncontrolled, high proliferation of plant tissue, widely referred to as plant tumour disease^4^,^5^. This is exemplified by the bacterium *Agrobacterium tumefaciens*, which induces crown gall tumour and is the most intensively studied plant tumour-inducing agent^6^. Many plant viruses are known to induce enation, tumour or gall formations in host plants; these types of viruses belong to the *Reoviridae*, *Luteoviridae*, *Geminiviridae* and *Tymoviridae* families^7^,^8^,^9^,^10^,^11^,^12^,^13^,^14^,^15^.

Rice black-streaked dwarf virus (RBSDV), a member of plant-infecting reoviruses of the genus *Fijivirus* (family *Reoviridae*), is currently widespread in China and is the causal agent of one of most destructive viral diseases in maize and rice in Asia^16^,^17^,^18^,^19^. RBSDV is transmitted by small brown planthoppers (*Laodelphax striatellus*) in a persistent, propagative manner. The mature virions of RBSDV are double shelled, with a fragile outer shell of 65–70 nm in diameter^20^. The RBSDV genome consists of 10 dsRNA segments 1801–4501 bp in length^18^,^21^. The genome segments 1, 2, 3, 4, 8 and 10 encode viral structural proteins P1, P2, P3, P4, P8 and P10, respectively, while segments 5, 6, 7 and 9 encode non-structural proteins P5, P6, P7-1 and P9-1, respectively. RBSDV replication and assembly occurs in viroplasm, which is formed by matrix protein P9-1^22^,^23^. RBSDV infection in maize causes rough dwarf diseases, characterised by plant dwarfing, vein clearing and formation of white tumours (enations) along the veins of the back sheathes of leaves^24^,^25^.

The structures and formations of plant tumours that are induced by pathogenic bacteria are highly characterised, but there has been relatively little information on those induced by plant viruses. A few structural studies on tumour tissue induced by RBSDV were mainly based on observation using high resolution electron microscopy^13^,^26^,^27^; therefore, the comprehensive view of the structural anatomy, cell composition and virus distribution of this highly proliferated tissue is still limited. Moreover, to gain insight into the physiological basis underlying the tumour formation, it is important to investigate the cellular proteins with differentially altered expression in the tumour tissue. To address the abovementioned issues, in this study, we performed both histological and proteomic analyses of tumour tissues in RBSDV-infected maize plants. Our analyses indicated a vascular origin of the tumour cells and identified several cellular proteins that are either up- or down-regulated in tumour tissues. Moreover, we observed that tumour tissues contain the highest accumulation of RBSDV. Finally, the significance of tumours as the multiplication sites of RBSDV is discussed.

## Results

### Elevated levels of *cdc2* gene transcripts in the tumour tissue

**Figure 1:**
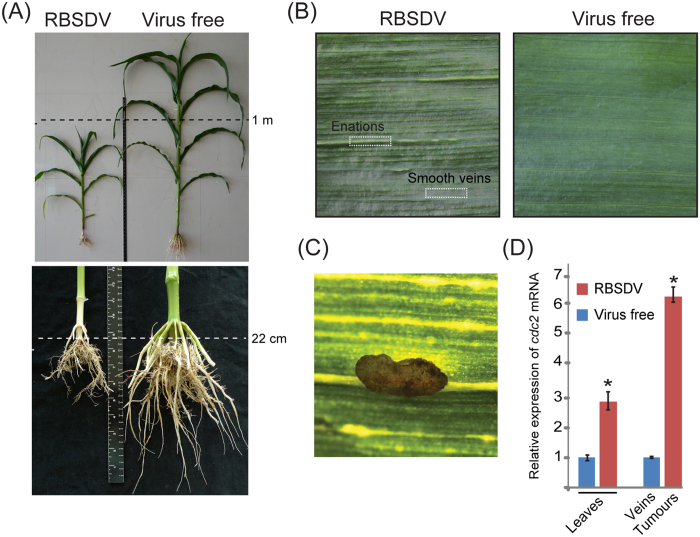
RBSDV symptoms in maize plants. (**A**) RBSDV-infected maize plant showing shoot and root stunting. (**B**) Enations along the veinal region on the abaxial side of infected leaves. (**C**) Tumour that has developed into a brown gall. (**D**) Steady-state levels of *cdc2* transcripts in leaves and tumours relative to those in virus-free tissues. *p ≤ 0.05 (student’s *t* test).

To obtain the experimental materials, maize plants infected with RBSDV were collected from field experimental plots, and the presence of the virus was confirmed by RT-PCR (data not shown). Compared to uninfected maize plants, the growth of RBSDV-infected plants was retarded, with the sizes of both shoots and roots being almost half those of uninfected plants (Fig. 1A). The leaves of RBSDV-infected plants developed streaked enlargements (enations) or white tumours along the veins (Fig. 1B), with some of those structures developing into brown galls, usually at the later stage of viral infection (Fig. 1C). First, we questioned whether the tumour formation is associated with regulation of the gene controlling cell division. *Cdc2* gene, which encodes a cyclin-dependent kinase (CDK), is central for cell cycle regulation^28^. Maize cdc2 has sequence homologies to human, *Schizosaccharomyces pombe* and *S. cerevisiae* cdc2 proteins and its transcripts were shown to be abundant in actively dividing tissues such as apical meristem and immature leaf^29^. Quantitative RT-PCR showed that the transcript accumulation level of the maize *cdc2* was around 6-fold higher in the tumour tissue than in veinal tissue of uninfected maize, while it was around 3-fold higher in infected leaves (whole laminae) than in virus-free leaves (Fig. 1D). This result suggests that tumour formation is associated with the reactivation of plant cell division.

### Up- and down-regulated proteins in the tumour tissue

**Figure 2:**
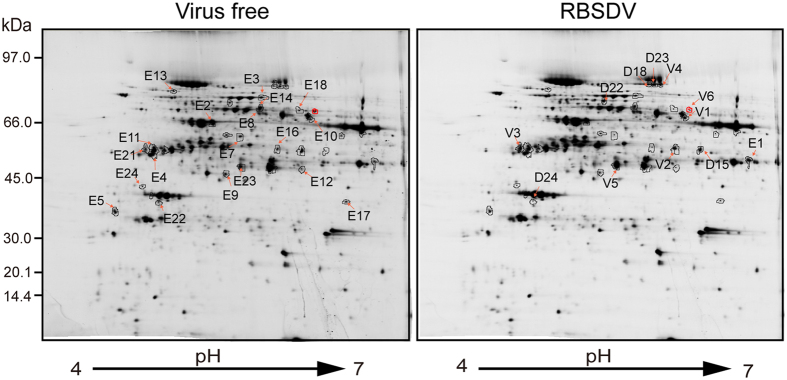
Proteomic analysis of differentially accumulated proteins in RBSDV-induced tumour tissue. Protein samples were separated in 2-DE gels and then stained with silver. The down- and up-regulated protein spots were labelled in the gel image of virus-free (vein) and RBSDV-infected (tumour) samples.

To further gain insight into the physiological changes that occur in the tumour tissue, we investigate the cellular proteins with altered accumulation in the tumour tissue induced by RBSDV using two-dimensional electrophoresis (2-DE) analysis comparing total proteins extracted from the tumour portion and vein of uninfected leaves. To compare the protein spots across gels, a match set was created from the images of the four gels with biological replicate samples. The 2-DE gel maps were stained with silver staining (Fig. 2), Cy-3 and Cy-5 fluorescent dyes (data not shown) and then subjected to analyses using different protein expression softwares. The analyses identified 38 protein spots with accumulation change between the tumour sample and uninfected samples (Fig. 2). Matrix-assisted laser desorption and ionisation time-of-flight mass spectrometry (MALDI-TOF MS) and database searches identified that seven protein spots were redundant proteins and that six protein spots were RBSDV-encoding proteins. Thus, there were 25 total individual maize proteins, of which 6 and 19 were up- and down-regulated, respectively (Table 1). Seventeen of these proteins have been annotated in the NCBI database with a putative function. Eight proteins are unknown; therefore, their functions are predicted by the presence of the conserved domains identified using the NCBI database. Based on their putative functions, those identified proteins can be classified into eight different functional categories, composed of photosynthesis, redox, energy pathways, amino acid synthesis, signalling, protein translation, carbohydrate metabolism and defence (Table 1). The largest group of identified proteins (9 proteins) belongs to the photosynthetic functional category, followed by 6 and 4 proteins belonging to the redox and energy pathway categories, respectively (Table 1). These results suggest that tumour formation in RBSDV-infected maize plants is associated with alteration of various plant metabolic pathways.

### Structure and cell composition of tumour tissue

**Figure 3:**
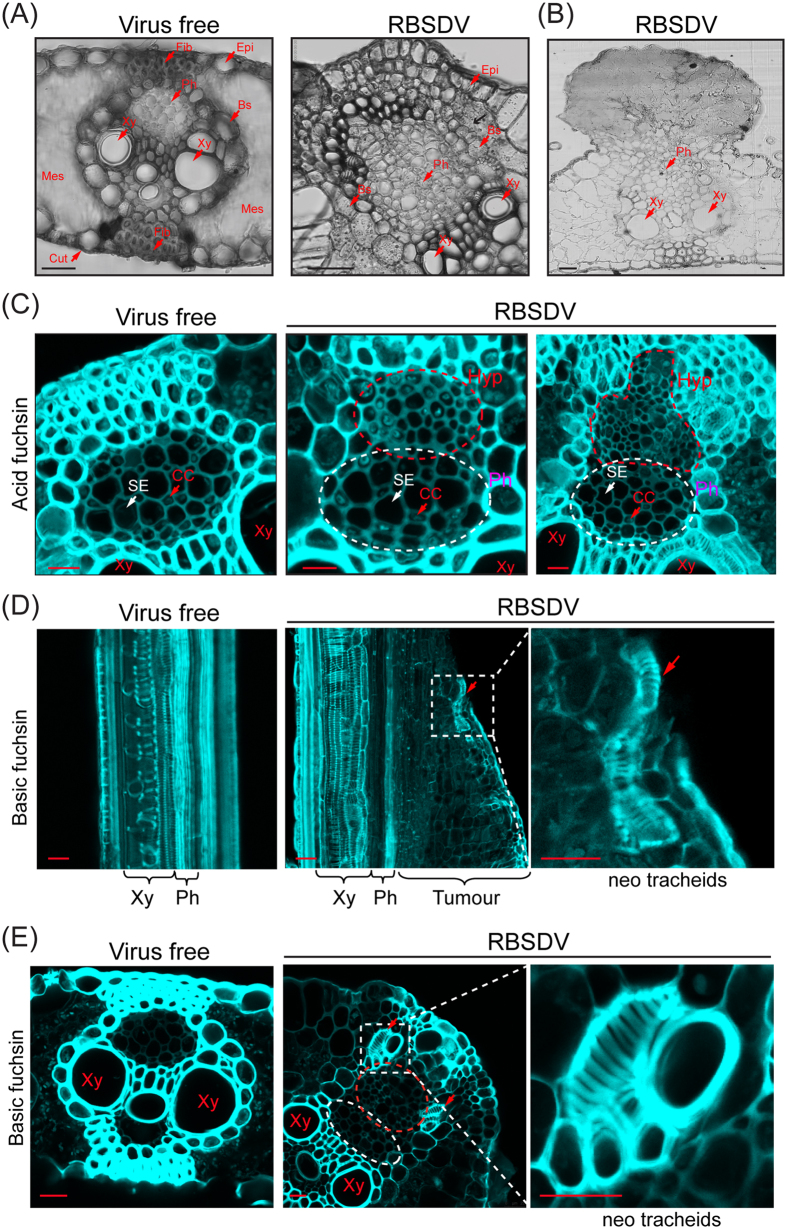
Internal structures of the RBSDV-induced tumour tissue. (**A,B**) Semi-thin cross-sections prepared from veinal region of virus-free leaf and enlarged leaf vein of RBSDV-infected plants (**A**) or brown galls (**B**). Xy, xylem; Ph, phloem; Bs, bundle sheath cells; Epi, epidermis; Fib, fibre tissue; Mes, mesophyll; Cut, cuticle. Bars, 50 μm. (**C**) Staining of a cross-section with acid fuchsin. SE, sieve element; CC, companion cell; Hyp, hyperplastic region. Bars, 20 μm. (**D**,**E**) Staining of the longitudinal sections (**D**) and cross-sections (**E**) with basic fuchsin. Bars, 20 μm.

Next, we carried out a histological analysis to characterise tumour structures induced by RBSDV infection. The transverse semi-thin sections of veinal regions of healthy leaves showed the typical tissue organisation of an annual maize leaf, which contains the cuticle, epidermis, mesophyll and vascular bundles (Fig. 3A). The vascular bundle mainly consists of xylem and phloem tissues and is enclosed by a single layer of sclerenchyma cells called bundle sheath cells as well as by supporting and protective tissues (fibre) (Fig. 3A). Observations of transverse sections from enlarged veins of RBSDV-infected leaves revealed an obvious different internal structure with highly proliferated cells. The most significant change is that the phloem tissues underwent hyperplasia and caused veinal enlargement (Fig. 3A). Bundle sheath cells were loosely arranged, having formed one or more layers, whereas they were tightly packaged into a single layer of sheath cells in healthy leaves. Those hyperplastic phloem tissues grew toward the lower epidermis and expanded beyond normal vascular boundaries, whereas the epidermal cells and xylem did not show abnormal proliferation (Fig. 3A). In one particular largely developed gall structure, the epidermis and cuticle layers were extruded and eventually were ruptured by the growth of tumour tissue (Fig. 3B).

Phloem tissue consists of sieve elements (SE) and companion cells (CC). Although both are alive, only CC have a nucleus and play a role in controlling the metabolism of sieve tubes^30^. To further examine the cell composition of those highly proliferated tissues, sections were stained with acid fuchsin, an acidic dye that stains the cytoplasm and nucleus. In the phloem of healthy leaf veins, SE (bigger and hollow) and CC (smaller and densely stained cells) are highly organised, in that each SE is adjoined by at least one CC (Fig. 3C). In contrast, the phloem area of the veins of infected leaves is composed of two regions: one with normal interlaced SE and CC arrangement, as observed in the veins of a healthy plant (Fig. 3C, white dashed line), and another with smaller, denser disorganised SE and nucleate cells, where multiple SEs are often arranged together without the adjacent CC (Fig. 3C, red dashed line). Thus, the latter is the neoplastic tissue that developed from the normal phloem tissue. These observations indicate that the tumour-like structures originated predominantly from hyperplastic development of phloem but with irregular structure and arrangement of the cells.

Several studies reported that rapidly growing plant tumours develop distinct vascular networks (neovascularisation) that provide nutrients and water for tumour growth^31^. To further characterise the tumour tissue induced by RBSDV infection, the sections were stained with basic fuchsin, which can stain lignified tracheary elements^32^,^33^. In longitudinal sections from virus-free samples, xylem structures (tracheids) were easily visualised using this staining method, as indicated by annular and helical forms of thickening (lignification) in vascular bundles (Fig. 3D). Interestingly, in the tumour tissue sections, tracheary-like structures that localise apart from the vascular bundle were observed within the neoplastic region (Fig. 3D, white dashed square). We also observed similarly such lignified vessel structures when transverse sections of tumour tissue were stained with basic fuchsin (Fig. 3E, white dashed square). Because those lignified vessels are not connected to the main vascular bundle, their function is unclear. Nevertheless, it is still possible that at a certain point during tumour growth, those treachery elements serve as conduits for passage of water or nutrients to developing tumour tissue.

### Cellular distribution of RBSDV

**Figure 4:**
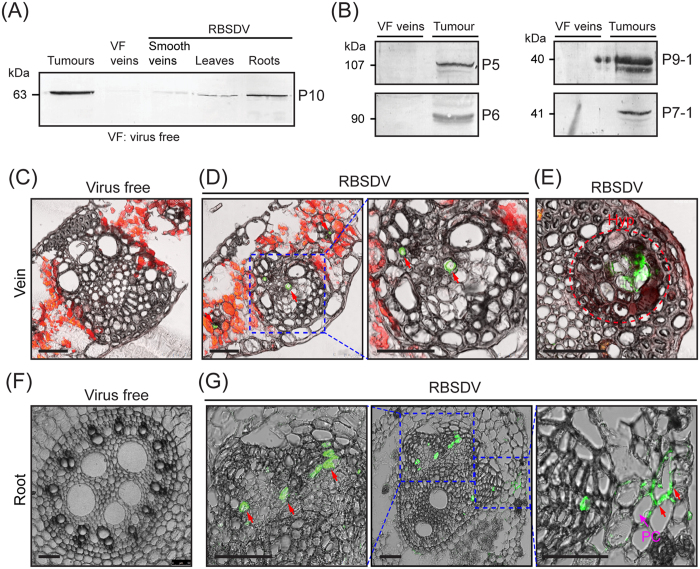
Cellular distribution of RBSDV in maize plants. (**A**) Western blot analysis of the accumulation of RBSDV P10 in tumours and other tissues. (**B**) Western blot analysis of the accumulation of RBSDV non-structural proteins in tumours. (**C**–**G**) Immunofluorescence detection of RBSDV in the cross-sections prepared from leaf veins (**C**) and roots (**F**) of non-infected plants or leaf veins (**D**), tumour tissue (**E**) and roots (**G**) of infected plants. Images are the merging of bright field and fluorescence images. The fluorescence signals are marked with red arrows. Hyp, hyperplastic region; PC, parenchyma cells. Bars, 50 μm.

To investigate RBSDV distribution in infected maize plant, we first analysed RBSDV accumulation in various tissues by western blotting using an antibody specific for the RBSDV outer coat protein (P10). Analysis showed that the accumulation of RBSDV coat protein was much more abundant in tumour tissue than in smooth veins, entire laminae (leaf blade) or roots of infected maize plants (Fig. 4A). P1 and P2, which are the other structural proteins of RBSDV, also were detected in the tumour tissue (data not shown). This result shows that tumour tissue contains the most abundant RBSDV.

To further examine the cellular distribution of RBSDV in infected maize plants, semi-thin sections were processed for immunofluorescence labelling using P10 antiserum. The fluorescent signals, indicating virus accumulation, were exclusively observed in phloem (Fig. 4D) and tumour regions (Fig. 4E). As a negative control, no fluorescence signal was detected in the section from uninfected samples (Fig. 4C) and in infected samples labelled with pre-immune serum (data not shown). In line with these observations, electron microscopy observation using ultra-thin sections prepared from leaf tissue of infected plants did not find RBSDV virions in the epidermal and mesophyll cells (data not shown). These results confirmed that RBSDV spread is confined to phloem and tumour tissues.

Because RBSDV accumulation was relatively higher in roots than in leaves (Fig. 4A), we also investigated the cellular distribution of RBSDV in roots by thin section and immunofluorescence labelling. Clearly, RBSDV infection drastically altered the internal structure of maize roots (Fig. 4F,G). There were fewer numbers of xylem vessels in the vascular bundles of infected samples than in those of uninfected samples. Similar to those of veins of infected plants, the vascular bundles contained some hyperplastic regions with disorganised cell arrangement and irregular cell structure. Virus accumulations were detected in the cells located in these hyperplastic regions (Fig. 4G). Thus, high levels of RBSDV accumulation in roots of maize are likely due to the development of hyperplastic cells in roots. Interestingly, florescent signals also were detected in adjacent parenchyma cells (Fig. 4G, PC), indicating that in roots of maize plants, RBSDV is not strictly confined to the phloem.

### Active replication of RBSDV in the tumour tissue

**Figure 5:**
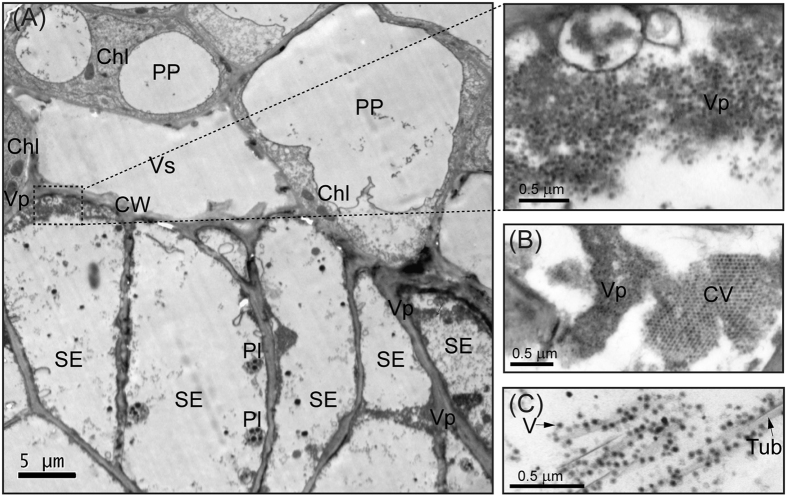
RBSDV-associated ultrastructures in tumour cells. (**A**–**C**) Electron micrograph of ultra-thin sections showing formation of viroplasm (**A**), crystalline arrays of virions (**B)** and tubular structures (**C**) in the cells. Chl, chloroplast; CV, crystalline arrays of virions; CW, cell wall; Pl, plastid; PP, phloem parenchyma; SE, sieve element; Tub, tubular structures; V, virion; Vp, viroplasm; Vs, vessel.

The presence of RBSDV non-structural viral proteins P5-1, P6, P7-1 and P9-1, which normally accumulate during virus replication, was identified by 2-DE analysis on tumour tissue (Table 1). In agreement with the 2-DE result, high accumulation levels of these non-structural viral proteins were also detected in the tumour tissue by western blotting (Fig. 4B). These results suggest that RBSDV replicates in the tumour tissues. To examine the presence of ultrastructures that are associated with RBSDV infection and replication in cells of tumour tissue, we carried out electron microscopy observations of ultra-thin sections. The formation of viroplasm structures (viral replication factories), indicated by the presence of amorphous, electron-dense cytoplasmic bodies with abundant numbers of RBSDV virions, was observed in SEs that were easily recognised by the presence of specific plastids and the absence of vacuoles (Fig. 5A). In addition, the presence of crystalline arrays of virions and tubular structures also was observed in these compartments (Fig. 5B,C). These observations further confirm that RBSDV indeed actively replicates in the tumour tissue.

## Discussion

Tumour formation is caused by the disturbance of normal control of cell division, resulting in reactivation and uncontrolled cell proliferation^5^. Cell cycle regulators control proper cell division and coordinate the transition between cell proliferation and differentiation stages. CDKs, in combination with the catalytic subunit called cyclin, play a central role in driving the mitotic cycle of eukaryotic cells. Briefly, entry into the cell cycle in the G_0_ phase is initiated by phosphorylation of the retinoblastoma (Rb) protein by the CDK and cyclin complex. This facilitates the release of the association of the Rb protein with the E2F and DP transcription factor complex, enabling this complex to regulate the transcriptional activation of several genes necessary for DNA replication, thus triggering the G_1_-to-S_1_ transition^34^,^35^,^36^. The perturbation of normal regulation of the cell cycle by plant virus infection has been well documented for DNA viruses belonging to the family Geminiviridae^37^. The geminivirus replication protein disrupts the Rb–E2F complex through the interaction with Rb protein^38^,^39^. Geminivirus infection also alters the transcriptional expression of a wide array of genes involved in cell cycle regulation^40^. In the case of viruses belonging to the genus *Curtovirus* in the family Geminiviridae, expression of the C2 or C4 protein was shown to up-regulate the transcriptional expression of cell cycle-related genes^41^,^42^. Note that the C4 protein is responsible for induction of enations in infected plants^9^. These studies clearly demonstrate that tumour formation by virus infection is associated with the disturbance of the regulation of the plant cell cycle. It is still not clear how RBSDV up-regulates *cdc2* gene transcription (Fig. 1D). One possibility is that one or more proteins encoded by RBSDV have activity to directly regulate the expression of host cell cycle-related genes. Because cell cycle initiation is stimulated by phytohormones, such as cytokinins and auxins^28^, RBSDV may induce the reactivation of cell division in maize plants through the stimulation of plant hormone production. Indeed, our analysis of rice plants infected with RBSDV showed an increase of indole-3-acetic acid levels and changes in other phytohormone levels (Liying Sun, unpublished results).

The effects of RBSDV infection on the expression of host cellular genes have been studied in rice and maize plants using proteomic and microarray analyses^43^,^44^,^45^, but those studies do not focus specifically on tumour tissue. Compared to those studies, our proteomic analysis identified much fewer numbers of proteins with altered expression, suggesting that a distinct regulation of gene expression occurs in the tumour tissue. Apparently, there are much more numbers of down-regulated than up-regulated proteins in the tumour tissue (19 versus 6) (Table 1). In particular, among those down-regulated proteins, many, including ribulose-1,5-bisphosphate carboxylase/oxygenase large subunit, fructose-1,6-bisphosphate aldolase, phosphoribulokinase and phosphoenolpyruvate carboxylase, are involved in photosynthetic processes (Table 1). This may suggest that cellular proteins are regulated so that tumour tissue could behave as a sink tissue. Furthermore, several enzymatic proteins with functions in redox reactions were found to be regulated in the tumour tissue. For example, accumulation of tyrosinase is around 4-fold reduced in the tumour tissue. Tyrosinase is a copper-containing enzyme that oxidises monophenols to *o*-diphenols^46^. In plants, it catalyses the production of pigments from tyrosine by oxidation, as seen in postharvest browning. Thus, the characteristic white colour of the tumour induced by RBSDV infection may be associated with reduced pigment biosynthesis. On the other hand, the accumulations of glyceraldehyde 3-phosphate dehydrogenase 2, cytosolic 2 (GAPC) and lipoxygenase 10 are around 1.5- and 1.7-fold more elevated, respectively. GAPC is essential in glycolysis and gluconeogenesis^47^ and is required for normal growth and development of plants^48^. Lipoxygenase catalyses the hydroperoxidation of lipids, containing a *cis*,*cis*-1,4-pentadiene structure^49^. In plants, this enzyme is involved in various aspects of plant physiology, including growth and development, defence against insect attack and response to abiotic stress^50^,^51^,^52^. We noted that no plant hormone-related protein was identified from our analysis, which is similar to the results of previous proteomic analyses of RBSDV-infected maize^44^, although a change in transcriptional level of hormone-related genes upon RBSDV infection in maize plants was detected using microarray analysis^43^. It is possible that 2-DE analysis is not sensitive enough to detect such changes. This likely also explains why cdc2 protein was not identified from 2-DE analysis. In summary, our proteomic analysis indicates that tumour formation induced by RBSDV infection is associated with various physiological and metabolic changes in the host plant.

The distribution profile of a virus in a plant host could be affected by various factors, including the suitability of specific tissues or cell types for virus replication, the ability of the virus to move between cells or tissues and the plant defence response activity^3^. It is interesting to point out that although the tumour-inducing plant viruses consist of diverse virus families with different genome properties and replication strategies (DNA or RNA), they are all similarly characterised with phloem-limited accumulations in their natural host plants. More importantly, they similarly induce hyperplastic development of phloem tissue^8^,^9^,^11^,^14^; therefore, virus accumulation and replication of those RNA viruses in the tumour tissue need to be analysed in great detail. In this study, immunofluorescence experiments showed that RBSDV exclusively accumulates in phloem and neoplastic cells (Fig. 4D,E). Moreover, viral non-structural proteins highly accumulated, and viroplasm formed in the neoplastic cells (Figs 4B and 5), providing strong evidence that the virus replicates in this tissue. It therefore seems plausible to assume that RBSDV alters cell fate and triggers cell division of specific vascular cells to provide a large amount of suitable cells for supporting efficient virus replication in the host plant. It is also interesting to investigate whether the tumour formation has a positive effect on the efficiency of viral transmission through the insect vector.

## Methods

### Viruses and plants

RBSDV-infected maize plants were obtained from an experimental field plot in Nanjing City, Jiangshu Province of China and provided by Dr. Zhao Han (Jiangsu Academy of Agricultural Sciences). Maize plants were grown on soil and naturally exposed to viruliferous small brown planthoppers.

### RNA extraction and quantitative RT-PCR

RNA was extracted with TRIzol (Invitrogen, USA) and further treated with DNAase RQ1 (Promega, USA). The first cDNA strands were synthesised using ReverTra Ace reverse transcriptase (Toyobo, Japan) with an oligo (dT) primer. The transcripts of maize *cdc2* (Accesion No. NM_001111872) were amplified using forward primer (5′-GTCCACAGCGAGAAGCGCATA-3′) and reverse primer (5′-CGCAACACCGTGGAGTATCTGGTA-3′). The 18S RNA of maize (Accession No. AF168884.1) was amplified using forward primer (5′-CCATCCCTCCGTAGTTAGCTTCT-3′) and reverse primer (5′-CCTGTCGGCCAAGGCTATATAC-3′) and used as an internal control. qRT-PCR was performed using the SYBR Green GoTaq qPCR master mix kit (Promega) on a CFX96TM Real-Time PCR Detection System apparatus (Bio-Rad). Four biological replicate samples were analysed.

### 2-DE and MALDI-TOF MS analyses

Each sample was pooled from six plants at the same pre-flowering stage. Protein samples were prepared as described previously^53^. Protein concentration was quantitative using a 2-D Quant Kit (GE Healthcare, USA) according to the manufacturer’s instructions and the amount of loaded protein samples was calculated. The resulting protein samples were then subjected to electrophoretic analysis using an optimised procedure for DIGE (GE Healthcare, UK) according to the manufacturer’s instructions. Prior to electrophoresis, protein samples were labelled using the CyDyes DIGE fluors (Cy3 and Cy5) (GE Healthcare). After electrophoresis, the 2-DE gels were visualised using an Ettan DIGE Imager (GE Healthcare). The gel images were transferred to the DeCyder 2D 7.0 software package (GE Healthcare) and further analysed with Differential In-gel Analysis modules (GE Healthcare). Protein level differences between two samples were analysed with the DeCyder-BVA (Biological Variation Analysis 5.02) module. MALDI-TOF-MS was performed by Shanghai Applied Protein Technology Co., Ltd. Briefly, silver stained protein spots were excised from the 2-DE gel and treated. The samples were then loaded on the MALDI target together with CHCA as a matrix for MALDI-TOF-MS analysis. All procedures were performed as described previously^54^. ProFound software was further used to identify protein spots from the NCBI non-redundant database via PMF experiments. All candidates with more than three identified CID spectra of peptides targeting the same protein and SEQUEST Xcorr values greater than 2.0 were further assessed by the comparison with Mr and Pi experimental values obtained from 2-DE. The sequences of identified proteins were further subjected to BLASTp to identify their homologous proteins and Blast2GO for functional annotation analysis.

### Semi-thin sectioning and chemical staining

Tissues (size of 40–50 mm) were embedded in low melting agarose and sectioned (30–50 μm thickness) using a Vibratom VT 1000 (Leica). Sections were examined with light microscopy (Leica). For chemical staining, sections were incubated in 0.3% acid fuchsin solution (w/v, in isopropanol) or 0.1% basic fuchsin solution (w/v, in distilled water, pH 8.5) for 2–5 min. The sections were observed using a confocal laser scanning microscope (TCS SP5, Leica), with excitation/emission filters of 488 nm/525–580 nm for acid fuschin and 514 nm/568–625 nm for basic fuschin.

### Western blot analysis

RBSDV P5, P6, P8, P9-1 and P7-1 antisera have been described previously^55^. The monoclonal antibody specific to RBSDV P10 was provided by Jianxiang Wu (Zhejiang University, China). Western blotting was performed as described previously^56^. For detection of P5-1, P6, P9-1 and P7-1, primary polyclonal antibodies (1:1,000) and secondary polyclonal alkaline phosphatase (AP)-conjugated goat anti-rabbit IgG (1:5,000) (Sigma) were used. P10 was detected using primary anti-P10 (1:1,000) and secondary AP-conjugated goat anti-mouse IgG (1:5,000) (Sigma-Aldrich).

### Immunofluorescence staining of semi-thin sections

Hand-cut tissues (about 30–50 mm thickness) were fixed with 4% paraformaldehyde and subsequently processed into 10–20-μm-thick sections with a cryostat (Leica CM1950), blocked in OCT then affixed to polylysine-coated glass slides. Tissue sections were incubated with the following antibodies: pre-immuno mouse antibody (1:500), mouse anti-P10 monoclonal antibody (1:500) and secondary antibody goat IgG anti-mouse conjugated to Fluorescein isothiocyanate (1:200) (Sigma-Aldrich). The fluorescent signals were visualised by a confocal laser scanning microscope with a laser excitation/emission filter of 488/505–555 nm.

### Electron microscopy

Preparation and electron microscopy observation of ultra-thin sections were carried out as described previously^55^,^57^.

## Additional Information

**How to cite this article**: Shen, J. *et al*. A phloem-limited fijivirus induces the formation of neoplastic phloem tissues that house virus multiplication in the host plant. *Sci. Rep*. **6**, 29848; doi: 10.1038/srep29848 (2016).

## Figures and Tables

**Table 1:** Up- and down regulated proteins in the tumour tissue induced by RBSDV infection.

Spot Code	Protein Name or Conserved domain	NCBI No.	Protein PI	Protein (kDa)	Protein score	Pep. count	Acc. fold
**Redox homeostasis**							
E14	NADP-malic enzyme	gi|30575690	6.2	70410.1	99.99	16	−2.01
E1	Glyceraldehyde-3-phosphate dehydrogenase 2, cytosolic 2	gi|162461501	6.41	36632.9	100	14	1.53
D18	Lipoxygenase10	gi|162464003	6.11	102481.2	100	37	1.77
E18	Tyrosinase*	gi|219885663	6.9	71507.9	100	20	−4.39
E5	Ferredoxin reductase	gi|224035923	5.57	19917.2	100	13	−1.54
E9	Ferredoxin reductase*	gi|194698944	7.55	41149.8	100	19	−1.58
**Amino acid synthesis**							
D15	Aspartate aminotransferase	gi|226508814	8.15	50547.8	100	27	2.87
E16	Glutamine synthetase	gi|195627092	7.64	47192.9	99.99	14	−2.1
**Signaling**							
E24	Unknown protein with PDZ domain*	gi|242043050	6.04	38162.7	88.96	12	−2.53
**Protein synthesis**							
E13	Elongation factor G*	gi|242076604	5.42	85338.9	100	30	−1.94
**Energy pathway**							
E8	ATP synthase CF1 alpha subunit	gi|260677417	6.03	55685.4	100	33	−1.56
E10	ATP synthase CF1 alpha subunit	gi|48478769	5.87	55747.4	100	31	−1.63
E21	ATP synthase beta subunit	gi|150035723	5.2	51106.7	100	18	−2.25
E2	Aconitase*	gi|238006740	6.76	55446.6	100	19	−1.51
**Photosynthesis**							
E11	Phosphoribulokinase	gi|226498182	5.84	45120.8	100	22	−1.69
E3	Transketolase	gi|75140229	5.47	73346.7	100	25	−1.51
E4	Sedoheptulose bisphosphatase1	gi|226506366	6.08	42303.3	100	21	−1.52
D24	Oxygen-evolving enhancer protein 1	gi|195619530	5.59	34782.7	100	23	1.53
E17	Ribulose-1,5-bisphosphate carboxylase/oxygenase large subunit	gi|290585776	6.99	26506.3	100	15	−2.14
E22	Ribulose-1,5-bisphosphate carboxylase/oxygenase large subunit	gi|11467200	6.33	53294.6	100	28	−2.39
E12	Fructose-1,6-bisphosphate aldolase*	gi|223975775	6.37	38407.5	100	24	−2.23
E23	Fructose-1,6-bisphosphate aldolase*	gi|226492201	7.63	41895.5	100	18	−2.45
D23	Phosphoenolpyruvate carboxylase	gi|27764449	5.73	109846.5	100	42	1.57
**Carbohydrate metabolism**							
D22	Phosphoglucomutase, cytoplasmic 1	gi|162463106	5.46	63285.9	100	24	1.7
**Defense**							
E7	Unknown protein with agglutinin domain*	gi|226531001	5.61	41336.1	100	18	−2.74
**Viral proteins**							
V1	P9-1[RBSDV]	gi|364506230	6.53	42388.2	100	25	
V2	P7-1[RBSDV]	gi|158967589	6.01	41003.6	100	19	
V3	P6 [RBSDV]	gi|20386793	4.97	89870.3	100	21	
V4	P10 [RBSDV]	gi|340748885	8.1	63156.3	100	13	
V5	P2 [RBSDV]	gi|337216535	6.04	141430.3	100	9	
V6	P5-1 [RBSDV]	gi|26279854	6.32	107015.1	100	3	

Pep., peptide; Acc., accumulation.

^*^Protein function is predicted by the presence of conserved domain.
